# The Utility of Real-Time Remote Auscultation Using a Bluetooth-Connected Electronic Stethoscope: Open-Label Randomized Controlled Pilot Trial

**DOI:** 10.2196/23109

**Published:** 2021-07-27

**Authors:** Takanobu Hirosawa, Yukinori Harada, Kohei Ikenoya, Shintaro Kakimoto, Yuki Aizawa, Taro Shimizu

**Affiliations:** 1 Department of Diagnostic and Generalist Medicine Dokkyo Medical University Tochigi Japan; 2 Docodemo Clinic Mashiko Tochigi Japan

**Keywords:** telemedicine, electronic stethoscope, simulator, remote auscultation, lung auscultation, cardiac auscultation, physical examination

## Abstract

**Background:**

The urgent need for telemedicine has become clear in the COVID-19 pandemic. To facilitate telemedicine, the development and improvement of remote examination systems are required. A system combining an electronic stethoscope and Bluetooth connectivity is a promising option for remote auscultation in clinics and hospitals. However, the utility of such systems remains unknown.

**Objective:**

This study was conducted to assess the utility of real-time auscultation using a Bluetooth-connected electronic stethoscope compared to that of classical auscultation, using lung and cardiology patient simulators.

**Methods:**

This was an open-label, randomized controlled trial including senior residents and faculty in the department of general internal medicine of a university hospital. The only exclusion criterion was a refusal to participate. This study consisted of 2 parts: lung auscultation and cardiac auscultation. Each part contained a tutorial session and a test session. All participants attended a tutorial session, in which they listened to 15 sounds on the simulator using a classic stethoscope and were told the correct classification. Thereafter, participants were randomly assigned to either the real-time remote auscultation group (intervention group) or the classical auscultation group (control group) for test sessions. In the test sessions, participants had to classify a series of 10 lung sounds and 10 cardiac sounds, depending on the study part. The intervention group listened to the sounds remotely using the electronic stethoscope, a Bluetooth transmitter, and a wireless, noise-canceling, stereo headset. The control group listened to the sounds directly using a traditional stethoscope. The primary outcome was the test score, and the secondary outcomes were the rates of correct answers for each sound.

**Results:**

In total, 20 participants were included. There were no differences in age, sex, and years from graduation between the 2 groups in each part. The overall test score of lung auscultation in the intervention group (80/110, 72.7%) was not different from that in the control group (71/90, 78.9%; *P*=.32). The only lung sound for which the correct answer rate differed between groups was that of pleural friction rubs (*P*=.03); it was lower in the intervention group (3/11, 27%) than in the control group (7/9, 78%). The overall test score for cardiac auscultation in the intervention group (50/60, 83.3%) was not different from that in the control group (119/140, 85.0%; *P*=.77). There was no cardiac sound for which the correct answer rate differed between groups.

**Conclusions:**

The utility of a real-time remote auscultation system using a Bluetooth-connected electronic stethoscope was comparable to that of direct auscultation using a classic stethoscope, except for classification of pleural friction rubs. This means that most of the real world’s essential cardiopulmonary sounds could be classified by a real-time remote auscultation system using a Bluetooth-connected electronic stethoscope.

**Trial Registration:**

UMIN-CTR UMIN000040828; https://tinyurl.com/r24j2p6s and UMIN-CTR UMIN000041601; https://tinyurl.com/bsax3j5f

## Introduction

Since the French physician René Laennec invented the stethoscope in 1816 [1], auscultation has been an essential component of clinical examination [2]. Auscultation is not only a highly cost-effective screening tool to detect abnormal clinical signs but also a useful means to build a good relationship between physician and patient. Although there has been a concerning decline in physicians’ auscultatory skills [2], auscultation will remain important in the 2020s, as cardiopulmonary diseases are important underlying or direct causes of death and morbidity, substantially impacting quality of life and health care costs [3,4].

However, auscultation became challenging during the COVID-19 pandemic. As medical staff also need to be protected from infection during an outbreak, the need for telemedicine is growing rapidly worldwide [5]. Therefore, remote auscultation should be developed for patients with acute or chronic cardiopulmonary diseases. However, several barriers exist for implementing telemedicine in clinical practice [6], including a lack of effectiveness via remote examination that can act as a substitute for direct physical examination. Auscultation is not one of the procedures typically mentioned in discussions of and research on telemedicine [2], which makes telemedicine seem impractical for patients with cardiopulmonary diseases.

Electronic stethoscopes are promising options to solve the problem of the lack of remote auscultation systems [7]. The electronic stethoscope not only is useful for remote auscultation but also has other advantages over the classic stethoscope. It can facilitate the differentiation of several types of cardiac and lung sounds by visualization of their sonograms during auscultation [2]; it can improve sound quality [8]; and it can contribute to better performance on auscultation, as personalized adjustments can be made [9]. Therefore, the electronic stethoscope can be used in telemedicine without a reduction in the quality of auscultation.

To the best of our knowledge, only a few studies have been conducted to determine the utility of real-time remote auscultation using an electronic stethoscope [7]. However, we are not aware of studies conducted for the direct comparison between real-time remote auscultation and direct auscultation. Therefore, in this study, we tested the hypothesis that the utility of real-time remote auscultation by a physician would be comparable to that of classical auscultation by a physician.

## Methods

### Study Design, Setting, and Participants

An open-label randomized controlled trial was designed to assess the utility of real-time remote auscultation using a Bluetooth-connected electronic stethoscope. Direct auscultation using a traditional stethoscope was used as control. To standardize and enhance the reliability of the assessment, we used a lung simulator for lung auscultation and a cardiology patient simulator for cardiac auscultation [10]. All sessions in this study were conducted at the skills laboratory at Dokkyo Medical University. As study participants, we recruited senior residents and faculty in the Department of Diagnostic and Generalist Medicine, as they were considered representative of general physicians working in community hospitals or clinics, the main population expected to perform auscultation in routine clinical practice. The only exclusion criterion was refusal to participate in this study. At baseline, no physicians exhibited hearing loss at their previous annual health checkup. This study was conducted in accordance with the current version of the Declaration of Helsinki. The study protocols were approved by the institutional ethics committee of Dokkyo Medical University, Tochigi, Japan (No. R-33-20J and R-37-19J). Written informed consent was obtained from all participants after a detailed explanation of the study and before participation.

### Procedures

#### Study Flow and Randomization

This study consisted of a lung auscultation part and a cardiac auscultation part. Each part contained a tutorial and a test session. Prior to the test session, all participants attended a tutorial session to become familiar with the device. Thereafter, participants were randomly assigned (simple randomization) to either the real-time remote auscultation group (intervention group) or the classical auscultation group (control group). The randomization was conducted separately in the lung and cardiac parts. Researchers were blinded in terms of allocation, by using a computer-generated allocation table to assign participants.

#### Tutorial Session

**Figure 1:**
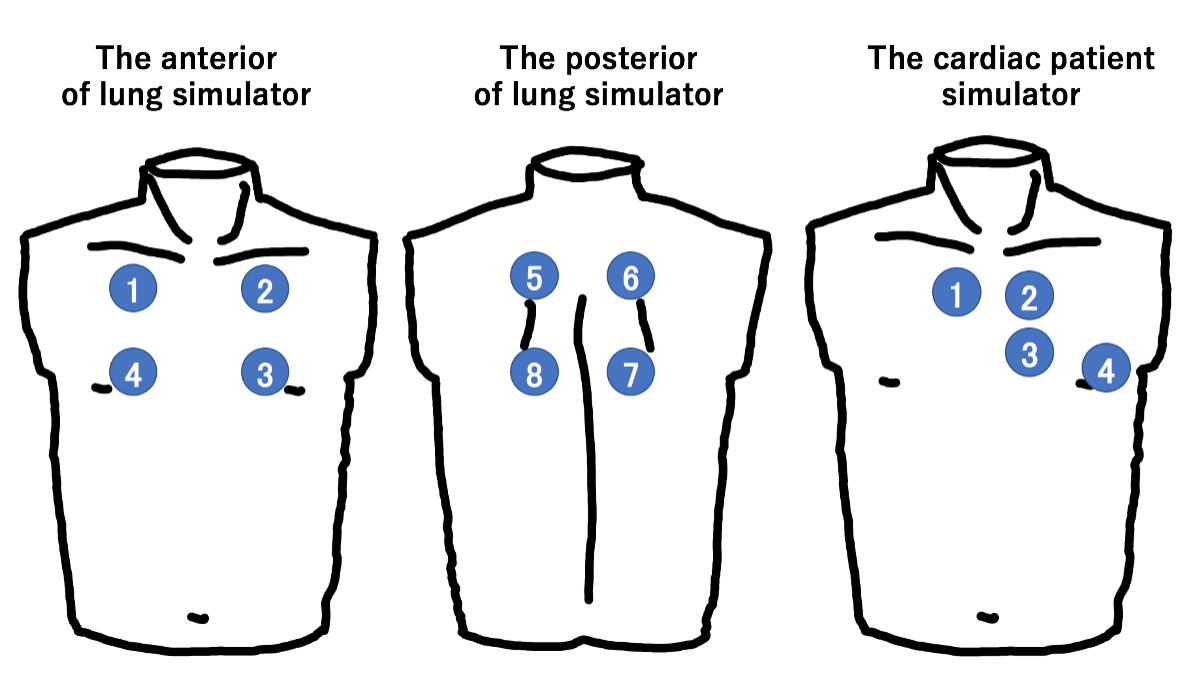
The 8 different areas of auscultation on the lung simulator and the 4 different areas of auscultation on the cardiac patient simulator.

In the tutorial sessions, all participants took part in auscultation using a traditional stethoscope (Littmann Cardiology III, 3M, St Paul, MN) on the lung simulator (Lung Sound Auscultation Trainer “LSAT” ver.2, model #MW28, Kyoto Kagaku Co, Ltd, Kyoto, Japan) and on the cardiac patient simulator (Cardiology Patient Simulator “K” ver.2, model #MW10, Kyoto Kagaku Co, Ltd). In each tutorial session, the participant listened to 15 sounds, with the correct classification being provided to participants. A short instruction for the simulator and the correct placements for auscultation were provided. In the tutorial session for lung auscultation, the following 15 sounds were played in a random order: normal lung sounds (standard, loud), wheezes (350-450 Hz, 600-700 Hz, 200-1000 Hz), 2 different rhonchi, stridor (twice), 2 different coarse crackles, 2 different fine crackles, and 2 different pleural friction rubs. In the tutorial session for cardiac auscultation, the following 15 sounds were played in random order: 3 different normal cardiac sounds (no S_2_ split, S_2_ split, and S_1_ split), 3 different third cardiac sounds (S_3_ gallop enhanced, S_3_ gallop, and S_3_-S_4_ gallop), aortic stenosis (twice), aortic regurgitation (twice), mitral regurgitation (3 times), mitral stenosis, and atrial fibrillation. Each participant was instructed to auscultate in the standardized positions on the simulators: 4 on the anterior and 4 on the posterior on the lung simulator; 4 on the cardiology patient simulator (Figure 1). Each sound was played for a maximum of 1 minute.

#### Test Session

**Figure 2:**
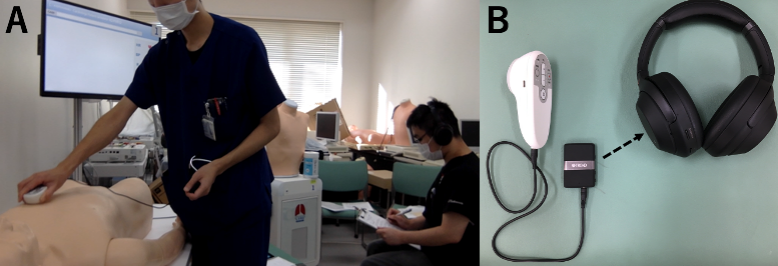
The remote auscultation processes: (A) The researcher is on the left, placing the electronic stethoscope on the cardiac patient simulator, and the participant is on the right listening to the cardiac sounds via a wireless, noise-canceling, stereo headset and Bluetooth transmitter; (B) the remote auscultation equipment including an electronic stethoscope; wireless, noise-canceling, stereo headset; and Bluetooth transmitter (Bluetooth connection is indicated with a dashed double arrow).

In the test sessions, participants in the intervention group auscultated all sounds remotely using an electronic stethoscope (JPES-01, MEMS CORE Co, Ltd, Miyagi, Japan), a Bluetooth transmitter and receiver (BT-DUO, TROND, Eastvale, CA), and a wireless, noise-canceling, stereo headset (WH-1000XM3, Sony Co, Tokyo, Japan), as depicted in Figures 2A and 2B. For this group, the researcher placed the electronic stethoscope and transmitter on the simulator, and participants could monitor the placement of the electronic stethoscope in real time. In the lung simulator, the LED panel on the side of the simulator indicated whether it was the inspiration or expiration phase. The monitoring screen of the cardiology patient simulator was modified to display only a heartbeat icon. Participants in the control group auscultated all sounds directly using the traditional stethoscope, placing it by themselves. For lung auscultation, of the 15 sounds played during the tutorial session, the following 10 sounds were played in a random order during the test session: normal lung sounds (normal, loud), wheezes (350-450 Hz, 600-700 Hz, 200-1000 Hz), rhonchi, stridor, coarse and fine crackles, and pleural friction rubs. For cardiac auscultation, of the 15 sounds played during the tutorial session, the following 10 sounds were played in random order during the cardiac test session: 2 different normal cardiac sounds (no S_2_ split, S_2_ split), 2 different S_3_ (S_3_ gallop enhanced, S_3_ gallop), aortic stenosis, aortic regurgitation, mitral regurgitation (twice), mitral stenosis, and atrial fibrillation. Auscultation positions and length were also the same as those in the tutorial session. During the test session, all participants filled in forms to indicate the types of sounds they recognized (Multimedia Appendix 1 and Multimedia Appendix 2).

### Lung Simulator

For lung auscultation, the same lung simulator (MW28) was used in the tutorial and test sessions. This simulator was designed for medical education training and includes 34 samples of lung sounds recorded from actual patients and reproduced using a high-quality sound system. These lung sounds were classified, according to the American Thoracic Society classification system, as continuous (wheezes, rhonchi, or stridor) or discontinuous (fine or coarse crackles) [11]. Fine crackles were defined as “relatively high-pitched sounds usually heard at the end of inspiration as air enters the acinar unit.” Coarse crackles were defined as “the low-pitched, bubbling sounds that result from the accumulation of secretions in larger bronchi and trachea” [11]. The default respiratory rate of 15 breaths/minute was used during the study.

### Cardiology Patient Simulator

For cardiac auscultation, the same cardiology patient simulator (MW10) was used in the tutorial and test sessions. This simulator was designed for medical education training and includes 88 cases of cardiac sounds recorded from actual patients and reproduced using a high-quality sound system.

### Electronic Stethoscope

The electronic stethoscope is equipped with pressure-sensitive sensors, and the signals are converted into sound waves. It is also equipped with a volume regulator and a frequency filter. The filter has a bell mode, diaphragm mode, and wide mode, which enhance the 20-100 Hz, 200-2000 Hz, and 20-2000 Hz frequency bands, respectively. In the lung and cardiac parts, we used the diaphragm mode and the bell mode, respectively. The transmitter transferred the sounds from the lung simulator to the headset via Bluetooth (A2DP: Advanced Audio Distribution Profile).

### Data Collection and Outcome Measures

Age, sex, and years since obtaining a degree in medicine were collected from all participants as baseline demographic data. All participants’ answers for each sound in the test session were collected. The primary outcome measure was the test score in each group. The rates of correct answers for each sound were the secondary outcome measures.

### Statistical Analysis

The correct answer in each group was compared using the Fisher exact test for primary and secondary outcome measures. Continuous variables for participant baseline characteristics are presented as medians (IQRs) and were compared using the Mann-Whitney *U* test. Categorical and binary variables for participant baseline characteristics are presented as numbers (percentages) and were compared using the Fisher exact test. A *P* value <.05 was considered statistically significant. All statistical tests were performed using R 3.6.0 for MacOS X (The R Foundation for Statistical Computing, Vienna, Austria).

## Results

### Participant Profiles

**Figure 3:**
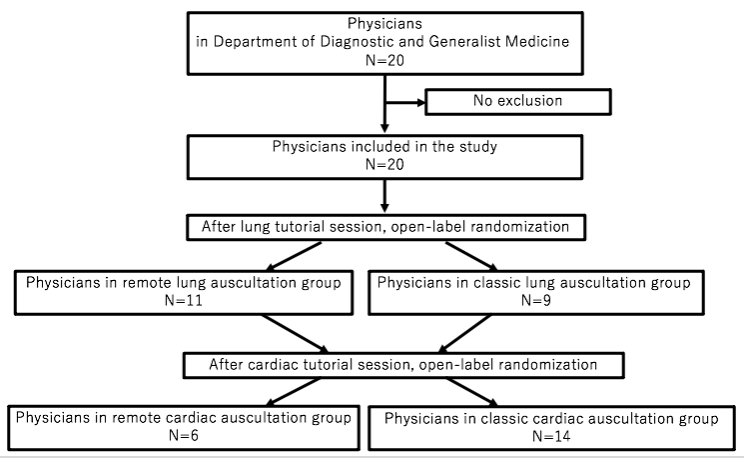
Flowchart of participant inclusion in the study.

In total, 20 physicians in the Department of Diagnostic and Generalist Medicine of Dokkyo Medical University were enrolled in the final analysis (Figure 3). Of these, 7 (35%) were senior residents (3-5 years after graduation), and 13 (65%) were faculty (≥6 years after graduation). The median age of all participants was 32 (IQR 8.5) years; 16 (16/20, 80%) were male, and the median time since graduation was 7.5 (IQR 7.5) years. In the lung part, 11 and 9 participants were assigned to the intervention and control groups, respectively. There were no statistically significant differences in participant age (*P*=.25), sex (*P*=.82), and years since graduation (*P*=.15) between the groups (Table 1). In the cardiac part, 6 and 14 participants were assigned to the intervention and control groups, respectively. There were no statistically significant differences in participant age (*P*=.99), sex (*P*=.99), and years since graduation (*P*=.78) between the groups (Table 1).

**Table 1:** Baseline characteristics of participants in the classical and remote cardiopulmonary auscultation groups.

	Lung auscultation			Cardiac auscultation		
Variable	Remote lung auscultation (n=11)	Classical lung auscultation (n=9)	*P* value	Remote cardiac auscultation (n=6)	Classical cardiac auscultation (n=14)	*P* value
Age (years), median (IQR)	34.0 (7.0)	29.0 (9.0)	.25^a^	32.5 (6.3)	32.5 (6.5)	.99^a^
Men, n (%)	9 (82)	7 (78)	.82^b^	6 (100)	11 (79)	.99^b^
Years after graduation (years), median (IQR)	10.0 (6.0)	4.0 (5.0)	.15^a^	8.0 (4.8)	7.4 (5.5)	.78^a^

^a^Mann-Whitney *U* test

^b^Fisher exact test.

### Diagnostic Performance

Test scores and rates of correct answers for each lung sound are summarized in Table 2. The total combined test score was 80/110 (72.7%) in the intervention group and 71/90 (78.9%) in the control group, with no differences between the groups (*P*=.32). There were no differences between the groups for normal lung sounds, wheezes, rhonchi, coarse crackles, fine crackles, or stridor. Only 3/11 (27%) participants in the intervention group correctly auscultated pleural friction rubs, whereas 7/9 (78%) in the control group did (*P*=.03).

Details of the answers for lung auscultation are supplied in Table 3 and Table 4. During exploratory data analysis, we determined that 4/22 (18%) instances of normal lung sounds were assigned as pleural friction rubs in the intervention group. On the other hand, 5/11 (45%) instances of pleural friction rubs were assigned as normal lung sounds in the same group.

**Table 2:** Lung sounds correctly identified.

Variable	Remote auscultation (n=11)	Classical auscultation (n=9)	*P* value^a^
Total, n/N (%)	80/110 (72.7)	71/90 (78.9)	.32
Normal, n/N (%)	16/22 (72.7)	18/18 (100)	.99
Wheezes, n/N (%)	25/33 (75.8)	19/27 (70.4)	.64
Rhonchi, n/N (%)	9/11 (81.8)	5/9 (55.6)	.21
Coarse crackles, n/N (%)	7/11 (63.6)	7/9 (77.8)	.50
Fine crackles, n/N (%)	9/11 (81.8)	6/9 (66.7)	.44
Pleural friction rubs, n/N (%)	3/11 (27.3)	7/9 (77.8)	.03
Stridor, n/N (%)	11/11 (100)	9/9 (100)	N/A^b^

^a^Fisher exact test.

^b^N/A: not applicable.

**Table 3:** Details of the participants’ answers for lung auscultation in the intervention (remote lung auscultation) group.

Correct answer	Normal	Wheezes	Rhonchi	Coarse crackles	Fine crackles	Pleural friction rubs	Stridor
Normal (n=22)	16	0	0	2	0	4	0
Wheezes (n=33)	2	25	3	0	0	1	2
Rhonchi (n=11)	0	1	9	0	0	0	1
Coarse crackles (n=11)	2	0	0	7	0	2	0
Fine crackles (n=11)	0	0	0	2	9	0	0
Pleural friction rubs (n=11)	5	1	0	2	0	3	0
Stridor (n=11)	0	0	0	0	0	0	11

**Table 4:** Details of the participants’ answers for lung auscultation in the control (traditional lung auscultation) group.

Correct answer	Normal	Wheezes	Rhonchi	Coarse crackles	Fine crackles	Pleural friction rubs	Stridor
Normal (n=18)	18	0	0	0	0	0	0
Wheezes (n=27)	0	19	6	0	0	0	2
Rhonchi (n=9)	0	2	5	0	0	0	2
Coarse crackles (n=9)	0	0	0	7	2	0	0
Fine crackles (n=9)	0	0	0	2	6	1	0
Pleural friction rubs (n=9)	1	0	1	0	0	7	0
Stridor (n=9)	0	0	0	0	0	0	9

Test scores and rates of correct answers for each cardiac sound are summarized in Table 5. The total combined test score was 50/60 (83.3%) in the intervention group and 119/140 (85.0%) in the control group, with no differences between the groups (*P*=.77). There were no differences between the groups for normal cardiac sounds, S_3_, aortic stenosis, aortic regurgitation, mitral stenosis, mitral regurgitation, and atrial fibrillation. Although not statistically significant, there was over a 30% difference in the score for mitral stenosis between the 2 groups: While 5 of 6 (84%) participants in the intervention group correctly auscultated, only 7 of 14 (50%) participants in the control group did (*P*=.19).

Details of the answers in the remote cardiac auscultation group are provided in Table 6. Misinterpretations between normal cardiac sounds and S_3_ sounds were frequently observed in the intervention group: 2 of 12 (17%) instances of normal cardiac sounds were assigned as S_3_ sounds. On the other hand, 4 of 12 (33%) instances of S_3_ sounds were assigned as normal cardiac sounds. Compared to the intervention group, participants in the control group made fewer misclassifications between normal cardiac sounds and S_3_ sounds (Table 7): Misclassifications of normal cardiac sound as S_3_ sounds occurred in 2 of 28 (7%) sounds, and misclassifications of S_3_ sounds as normal cardiac sounds occurred in 5 of 28 (17%) sounds. On the other hand, misinterpretations between mitral stenosis sounds and mitral regurgitation sounds were frequently observed in the control group: 6 of 14 (43%) instances of mitral stenosis sounds were assigned as mitral regurgitation sounds. In comparison, 4 of 28 (14%) instances of mitral regurgitation sounds were assigned as mitral stenosis sounds.

**Table 5:** Cardiac sounds correctly identified.

Variable	Remote auscultation (n=6)	Classical auscultation (n=14)	*P* value^a^
Total, n/N (%)	50/60 (83.8)	119/140 (85.0)	.77
Normal, n/N (%)	9/12 (75.0)	26/28 (92.9)	.14
S_3_, n/N (%)	8/12 (66.7)	22/28 (78.6)	.43
Aortic stenosis, n/N (%)	5/6 (83.3)	14/14 (100)	.99
Aortic regurgitation, n/N (%)	6/6 (100)	13/14 (92.9)	.99
Mitral stenosis, n/N (%)	5/6 (83.8)	7/14 (50.0)	.19
Mitral regurgitation, n/N (%)	11/12 (91.7)	23/28 (82.1)	.45
Atrial fibrillation, n/N (%)	6/6 (100)	14/14 (100)	N/A^b^

^a^Fisher exact test.

^b^N/A: not applicable.

**Table 6:** Details of the participants’ answers for cardiac auscultation in the intervention (remote cardiac auscultation) group.

Correct answer	Normal	S_3_	Aortic stenosis	Aortic regurgitation	Mitral stenosis	Mitral regurgitation	Atrial fibrillation
Normal (n=12)	9	2	0	0	1	0	0
S_3_ (n=12)	4	8	0	0	0	0	0
Aortic stenosis (n=6)	0	0	5	0	0	1	0
Aortic regurgitation (n=6)	0	0	0	6	0	0	0
Mitral stenosis (n=6)	0	0	0	0	5	1	0
Mitral regurgitation (n=12)	0	0	0	0	1	11	0
Atrial fibrillation (n=6)	0	0	0	0	0	0	6

**Table 7:** Details of the participants’ answers for cardiac auscultation in the control (traditional cardiac auscultation) group.

Correct answer	Normal	S_3_	Aortic stenosis	Aortic regurgitation	Mitral stenosis	Mitral regurgitation	Atrial fibrillation
Normal (n=28)	26	2	0	0	0	0	0
S_3_ (n=28)	5	22	0	0	1/28	0	0
Aortic stenosis (n=14)	0	0	14	0	0	0	0
Aortic regurgitation (n=14)	0	0	0	13	1	0	0
Mitral stenosis (n=14)	0	0	0	1	7	6	0
Mitral regurgitation (n=28)	0	0	1	0	4	23	0
Atrial fibrillation (n=14)	0	0	0	0	0	0	14

## Discussion

### Principal Findings

In this study, there were 4 main findings. First, using a simulator, we demonstrated that the utility of real-time remote auscultation using a Bluetooth-connected electronic stethoscope was comparable to that of direct auscultation using a classic stethoscope. From previous finding of lung auscultation, coarse crackles, fine crackles, wheezes, and stridor are useful for diagnosing bronchitis or pneumonia [12-14], interstitial pulmonary fibrosis or pneumonia [12,15-19], exacerbation of asthma or chronic obstructive pulmonary disease [12,19], and upper-airway obstruction [19,20], respectively. For cardiac auscultation, valvular cardiac diseases and irregular rhythm disease were not misclassified as normal cardiac sounds in real-time remote auscultation and classical groups. The correct detection of S_3_ is also an essential skill in diagnosing various cardiac diseases such as congestive heart failure, ischemic heart disease, cardiomyopathies, myocarditis, cor pulmonale, high-output states, left-to-right intracardiac shunts, and complete atrioventricular block [21]. Therefore, the results of this study suggest that real-time remote auscultation would be useful for the diagnosis of these diseases. At the bedside, this would allow medical staff to screen patients for adventitious sounds at a distance, protecting themselves from infectious diseases such as COVID-19.

Second, the rate of correct answers for pleural friction rubs was lower in the real-time remote lung auscultation group than in the direct lung auscultation group. Therefore, it would be challenging to diagnose pleuritis with real-time remote lung auscultation [12].

Third, in the real-time remote lung auscultation group, we observed a trend for confusion of normal lung sounds and pleural friction rubs. Respecting pleural friction rubs, 45% were misclassified as normal lung sounds in the remote auscultation group in this study. According to the participants, placement of the electronic stethoscope to the surface of the lung simulator caused a bit of noise. Electronic stethoscopes are sensitive to electronic and ambient noise, and this placement noise may be the cause of the difficulty observed in auscultation of pleural friction rub.

Fourth, the rate of correct answers for mitral stenosis was higher in the real-time remote cardiac auscultation than in the direct cardiac auscultation group. According to the participants, the monitoring screen of the simulator was on the caudal side. The screen showed a heartbeat icon regardless of whether it was the systolic or diastolic phase without any waveform. In the direct cardiac auscultation group, it was difficult to watch a display with auscultation. On the other hand, in the real-time remote auscultation group, the participants could watch the screen to detect the systolic or diastolic phase with auscultation. This may have been the cause for the misinterpretations between mitral stenosis sounds and mitral regurgitation sounds in the direct auscultation group.

### Strengths

There were 3 strengths of note in this study. First, use of simulators allowed us to gather standardized data, reducing bias. Second, the direct comparison of a novel auscultation technology with classical auscultation as control adds value to this randomized controlled study. Third, all participants were physicians who specialized in general internal medicine. Thus, the study participants were representative of general physicians working in community hospitals or clinics, the main population expected to perform auscultation in routine clinical practice.

### Limitations

This study was a pilot study and had several limitations. First, the sample size was small and did not include real patient data. Fully powered trials need to be conducted to better show equivalence. There was grouping variation through simple randomization, especially in the cardiac part, with 6 in the intervention group vs 14 in the control group. This grouping variation may have affected the detection power in this study. Therefore, in future studies, the efficacy of real-time remote auscultation has to be confirmed at the bedside with a larger sample size. Second, as the researcher who placed the electronic stethoscope on the simulators could not hear the sounds while doing so, the timing of the change in auscultation position could not be adjusted for optimum auscultation. Participants in the classical auscultation group were able to change the auscultation position on their own, which may have given this group an advantage over the remote auscultation group. Third, the respiratory rate and phase of crackle could not be adjusted with the lung simulator. The default respiratory rate was slower than that of real patients with respiratory diseases. Therefore, the results from this study may not be generalizable to auscultation at the bedside. Fourth, in this study, the technique used for classical auscultation was not standardized. This may have limited the reproducibility of our results. Fifth, there might be some dependences among answers within-subject.

### Comparison With Prior Work

To the best of our knowledge, there has been no other study in which real-time remote auscultation and classical auscultation were directly compared. In terms of the accuracy of classical auscultation using the simulator, our results, which showed variable accuracy depending on the type of sounds, were consistent with previous studies [10,22]. In both previous studies, the total accuracies varied from 62% to 89.7%, which may have depended on the difficulty of tests. For example, one study that showed a low accuracy rate included 3 types of S_2_, S_4_, and S_3_+S_4_, whereas another study that showed a high accuracy rate only included S_2_ split, mitral regurgitation, and aortic stenosis. Regarding the difficulty of testing, our study may be close to the previous 2 studies.

In a previous study of the accuracy of identifying lung sounds using classical auscultation, stridor was not included in the evaluation [10]. Stridor can be identified without a stethoscope. However, we included stridor in this study, as its detection with electronic stethoscopes was reported in other studies [23].

We are aware of 1 study reporting results regarding the utility of real-time remote auscultation [7]. In that study, the interobserver concordance of remote auscultation via the internet (93.7%-94.0%) was lower than that of direct auscultation (98.4%-98.9%), but not statistically significant. Although remote auscultation in that study was not conducted in real time, the results are similar to ours in terms of a slightly lower accuracy in the remote auscultation group than in the conventional auscultation group.

### Conclusions

This study demonstrates that the utility of a Bluetooth-connected, real-time remote auscultation system is comparable to that of classical, direct auscultation, except for pleural friction rubs. Future studies focused on real-time auscultation through Wi-Fi or the internet are warranted. Furthermore, this study leads the way for further studies in real patients with fully powered trials. In a future study, visualized waves of sounds [24] or artificial intelligence [25] would be supported to detect abnormal sounds.

## Appendix

Multimedia Appendix 1 The format of the questionnaire for identification of the 10 lung sounds.

Multimedia Appendix 2 The format of the questionnaire for identification of the 10 cardiac sounds.

Multimedia Appendix 3 CONSORT-eHEALTH checklist (V 1.6.1).
