# Supplementary material for: The Utility of Real-Time Remote Auscultation Using a Bluetooth-Connected Electronic Stethoscope: Open-Label Randomized Controlled Pilot Trial
Source: JMIR Mhealth Uhealth. 2021 Jul 27;9(7):e23109. doi: 10.2196/23109 (PMC8367161; doi:10.2196/23109)
Supplement: Multimedia Appendix 1 [file mhealth_v9i7e23109_app1.pdf]

The questionnaires of the following study: The Utility of Real-Time Remote Lung auscultation Using a Bluetooth-Connected Electronic Stethoscope: Open-Label Randomized Controlled Trial

Data ______________________ Gender ______________________

Age ______________________ Years since graduation ______________________

Auscultation type: Classical auscultation / Remote auscultation

1. ☐ Normal ☐ Wheezes ☐ Stridor ☐ Rhonchi
   ☐ Fine crackle ☐ Coarse crackle ☐ Pleural rubs
2. ☐ Normal ☐ Wheezes ☐ Stridor ☐ Rhonchi
   ☐ Fine crackle ☐ Coarse crackle ☐ Pleural rubs
3. ☐ Normal ☐ Wheezes ☐ Stridor ☐ Rhonchi
   ☐ Fine crackle ☐ Coarse crackle ☐ Pleural rubs
4. ☐ Normal ☐ Wheezes ☐ Stridor ☐ Rhonchi
   ☐ Fine crackle ☐ Coarse crackle ☐ Pleural rubs
5. ☐ Normal ☐ Wheezes ☐ Stridor ☐ Rhonchi
   ☐ Fine crackle ☐ Coarse crackle ☐ Pleural rubs
6. ☐ Normal ☐ Wheezes ☐ Stridor ☐ Rhonchi
   ☐ Fine crackle ☐ Coarse crackle ☐ Pleural rubs
7. ☐ Normal ☐ Wheezes ☐ Stridor ☐ Rhonchi
   ☐ Fine crackle ☐ Coarse crackle ☐ Pleural rubs
8. ☐ Normal ☐ Wheezes ☐ Stridor ☐ Rhonchi
   ☐ Fine crackle ☐ Coarse crackle ☐ Pleural rubs
9. ☐ Normal ☐ Wheezes ☐ Stridor ☐ Rhonchi
   ☐ Fine crackle ☐ Coarse crackle ☐ Pleural rubs
10. ☐ Normal ☐ Wheezes ☐ Stridor ☐ Rhonchi
    ☐ Fine crackle ☐ Coarse crackle ☐ Pleural rubs

Memo

