# Supplementary material for: The Utility of Real-Time Remote Auscultation Using a Bluetooth-Connected Electronic Stethoscope: Open-Label Randomized Controlled Pilot Trial
Source: JMIR Mhealth Uhealth. 2021 Jul 27;9(7):e23109. doi: 10.2196/23109 (PMC8367161; doi:10.2196/23109)
Supplement: Multimedia Appendix 2 [file mhealth_v9i7e23109_app2.pdf]

The questionnaires of the following study: The Utility of Real-Time Remote Cardiac auscultation Using a Bluetooth-Connected Electronic Stethoscope: Open-Label Randomized Controlled Trial

Data ______________________ Gender ______________________

Age ______________________ Years since graduation ______________________

Auscultation type: Classical auscultation / Remote auscultation

1. ☐ Normal ☐ S3 ☐ Aortic Stenosis (AS) ☐ Aortic Regurgitation (AR)
   ☐ Mitral Stenosis (MS) ☐ Mitral Regurgitation (MR) ☐ Atrial Fibrillation (Af)

2. ☐ Normal ☐ S3 ☐ Aortic Stenosis (AS) ☐ Aortic Regurgitation (AR)
   ☐ Mitral Stenosis (MS) ☐ Mitral Regurgitation (MR) ☐ Atrial Fibrillation (Af)

3. ☐ Normal ☐ S3 ☐ Aortic Stenosis (AS) ☐ Aortic Regurgitation (AR)
   ☐ Mitral Stenosis (MS) ☐ Mitral Regurgitation (MR) ☐ Atrial Fibrillation (Af)

4. ☐ Normal ☐ S3 ☐ Aortic Stenosis (AS) ☐ Aortic Regurgitation (AR)
   ☐ Mitral Stenosis (MS) ☐ Mitral Regurgitation (MR) ☐ Atrial Fibrillation (Af)

5. ☐ Normal ☐ S3 ☐ Aortic Stenosis (AS) ☐ Aortic Regurgitation (AR)
   ☐ Mitral Stenosis (MS) ☐ Mitral Regurgitation (MR) ☐ Atrial Fibrillation (Af)

6. ☐ Normal ☐ S3 ☐ Aortic Stenosis (AS) ☐ Aortic Regurgitation (AR)
   ☐ Mitral Stenosis (MS) ☐ Mitral Regurgitation (MR) ☐ Atrial Fibrillation (Af)

7. ☐ Normal ☐ S3 ☐ Aortic Stenosis (AS) ☐ Aortic Regurgitation (AR)
   ☐ Mitral Stenosis (MS) ☐ Mitral Regurgitation (MR) ☐ Atrial Fibrillation (Af)

8. ☐ Normal ☐ S3 ☐ Aortic Stenosis (AS) ☐ Aortic Regurgitation (AR)
   ☐ Mitral Stenosis (MS) ☐ Mitral Regurgitation (MR) ☐ Atrial Fibrillation (Af)

9. ☐ Normal ☐ S3 ☐ Aortic Stenosis (AS) ☐ Aortic Regurgitation (AR)
   ☐ Mitral Stenosis (MS) ☐ Mitral Regurgitation (MR) ☐ Atrial Fibrillation (Af)

10. ☐ Normal ☐ S3 ☐ Aortic Stenosis (AS) ☐ Aortic Regurgitation (AR)
    ☐ Mitral Stenosis (MS) ☐ Mitral Regurgitation (MR) ☐ Atrial Fibrillation (Af)
